# High-definition transcranial direct current stimulation (HD-tDCS) as augmentation therapy in late-life depression (LLD) with suboptimal response to treatment—a study protocol for a double-blinded randomized sham-controlled trial

**DOI:** 10.1186/s13063-022-06855-z

**Published:** 2022-10-28

**Authors:** Sze Ting Joanna Ngan, Lap Kei Chan, Wai Chi Chan, Linda Chiu Wa Lam, Wan Kei Li, Kelvin Lim, Ego Or, Pui Fai Pang, Ting Keung Poon, Mei Cheung Mimi Wong, Ying King Anna Wu, Pak Wing Calvin Cheng

**Affiliations:** 1 New Clinical Building, Queen Mary Hospital, 2/F, 102 Pok Fu Lam Road, Pok Fu Lam, Hong Kong, Hong Kong; 2 Department of Psychiatry, Kwai Chung Hospital, Kwai Chung, Hong Kong, Hong Kong; 3 Department of Psychiatry, Tai Po Hospital, G/F, Multi-Centre, Tai Po, Hong Kong, Hong Kong; 4 Department of Psychiatry, University of Minnesota Medical School, Minneapolis, MN USA; 5 Department of Psychiatry, United Christian Hospital, Kwun Tong, Hong Kong, Hong Kong; 6 Department of Psychiatry, Kowloon Hospital, Kadoorie Hill, Hong Kong, Hong Kong; 7 Department of Psychiatry, Pamela Youde Nethersole Eastern Hospital, Chai Wan, Hong Kong, Hong Kong

**Keywords:** High-definition transcranial direct current stimulation, Augmentation therapy, Late-life depression

## Abstract

**Background:**

Late-life depression (LLD) has a poorer prognosis and higher relapse rate than younger adults, with up to one third of patients with LLD showing suboptimal response to antidepressant therapy. LLD has been associated with significant impairment in cognition and daily functioning. Few studies have evaluated the therapeutic effects of high-definition transcranial direct current stimulation (HD-tDCS) on depressive and cognitive symptoms of LLD. The current randomized controlled trial assesses the efficacy of HD-tDCS as an augmentation therapy with antidepressants compared to sham-control in subjects with LLD.

**Methods:**

Fifty-eight patients with LLD will be recruited and randomly assigned to the active HD-tDCS or sham HD-tDCS group. In both groups, patients will receive the active or sham intervention in addition to their pre-existing antidepressant therapy, for 2 weeks with 5 sessions per week, each lasting 30 min. The primary outcome measures will be the change of depressive symptoms, clinical response and the remission rate as measured with the Hamilton Depression Rating scale (HAMD-17) before and after the intervention, and at the 4th and 12th week after the completed intervention. Secondary outcome measures include cognitive symptoms, anxiety symptoms, daily functioning and adverse effects.

**Discussion:**

Older adults with depression are associated with poorer outcomes or unsatisfactory responses to antidepressant therapy, and significant cognitive decline. Therefore, a new effective treatment option is needed. This randomized control trial aims at assessing the efficacy of HD-tDCS on ameliorating the depressive, cognitive and anxiety symptoms, and improving the daily functioning of subjects with LLD.

**Trial registration:**

ClinicalTrials.gov NCT05322863. Registered on 11 April 2022.

**Supplementary Information:**

The online version contains supplementary material available at 10.1186/s13063-022-06855-z.

## Administrative information

Note: the numbers in curly brackets in this protocol refer to the SPIRIT checklist item numbers. The order of the items has been modified to group similar items (see http://www.equator-network.org/reporting-guidelines/spirit-2013-statement-defining-standard-protocol-items-for-clinical-trials/).

**Table:** 

Title {1}	High-definition transcranial direct current stimulation (HD-tDCS) as augmentation therapy in late-life depression (LLD) with suboptimal response to treatment—a study protocol for a double-blinded randomized sham-controlled trial
Trial registration {2a and 2b}.	ClinicalTrials.gov ID NCT05322863 released on 11 April 2022 https://clinicaltrials.gov/ct2/show/NCT05322863
Protocol version {3}	23 February, 2022 Version n˚1.
Funding {4}	This study is sponsored by the Health and Medical Research Fund (HMRF) (Reference number: 17181141).
Author details {5a}	Miss. Sze Ting Joanna NGAN Institutional address: 2/F, New Clinical Building, Queen Mary Hospital, 102 Pok Fu Lam Road, Pok Fu Lam, Hong Kong Email address: jojongan@hku.hk Dr. Lap Kei CHAN Institutional address: Department of Psychiatry, Kwai Chung Hospital, Hong Kong Email address: clk793@ha.org.hk Dr. Wai Chi CHAN Institutional address: 2/F, New Clinical Building, Queen Mary Hospital, 102 Pok Fu Lam Road, Pok Fu Lam, Hong Kong Email address: waicchan@hku.hk Prof. Linda Chiu Wa LAM Institutional address: G/F, multi-centre, Department of Psychiatry, Tai Po Hospital, Hong Kong Email address:cwlam@cuhk.edu.hk Miss. Wan Kei LI Institutional address: 2/F, New Clinical Building, Queen Mary Hospital, 102 Pok Fu Lam Road, Pok Fu Lam, Hong Kong Email address: u3548669@connect.hku.hk Prof. Kelvin LIM Institutional address: Department of Psychiatry, University of Minnesota Medical School, Minnesota, United States Email address: kolim@umn.edu Mr. Ego OR Institutional address: 2/F, New Clinical Building, Queen Mary Hospital, 102 Pok Fu Lam Road, Pok Fu Lam, Hong Kong Email address: egoor@connect.hku.hk Dr. Pui Fai PANG Institutional address: Department of Psychiatry, United Christian Hospital, Hong Kong Email address: edwinpfpang@hotmail.com Dr. Ting Keung POON Institutional address: Department of Psychiatry, Kowloon Hospital, Hong Kong Email address: poontk@ha.org.hk Dr. Mei Cheung Mimi WONG Institutional address: Department of Psychiatry, United Christian Hospital, Hong Kong Email address: mimi.mimiwong@gmail.com Dr. Ying King Anna WU Institutional address: Department of Psychiatry, Pamela Youde Nethersole Eastern Hospital, Hong Kong Email address: wuyka@ha.org.hk Dr. Pak Wing Calvin CHENG Institutional address: 2/F, New Clinical Building, Queen Mary Hospital, 102 Pok Fu Lam Road, Pok Fu Lam, Hong Kong Email address: chengpsy@hku.hk
Name and contact information for the trial sponsor {5b}	N/A, no sponsors are included in this study
Role of sponsor {5c}	N/A, no sponsors are included in this study

## Introduction

### Background and rationale {6a}

#### Late-life depression (LLD) has a great impact on the health system

Most of the developed countries are facing a significant challenge of having a significant proportion of their ageing population suffer from depression in which the burden of LLD will continue to increase. LLD, which refers to adults who are 60 years or older, is associated with a poorer long-term prognosis, a more chronic course, and a higher relapse rate than that in younger adults [1]. The treatment response to medication is unsatisfactory. Fewer than 50% of patients with LLD achieve symptomatic remission [2]. Therefore, a new treatment option that is effective and well tolerated by older adults is desperately needed.

#### Alternative treatment options

Transcranial direct-current stimulation (tDCS) is a non-invasive method of neurostimulation in which a weak, direct electric current (amplitude < 2 mA) is applied through electrodes placed on the scalp. It is portable, relatively inexpensive and easy to use. It is also a relatively safe intervention tool with common adverse effects largely limited to the stimulation site including itchiness, redness, tingling and burning sensation and possible transient headache [3]. There are no severe adverse events recorded in more than 40 previous sham-controlled studies of older adults [3]. It exerts its treatment effect by inducing changes in neuronal activity and by modulating synaptic release probability uptake and sensitivity [4]. tDCS enhances long-term plasticity and alters the rate of neurotransmitter release [4]. Its effects have been reported up to an hour after stimulation, which is essential to achieve a persistent treatment effect [5]. One of the main neurological deficits in depression is the serotonin deficit and hypoactivity of the left dorsolateral prefrontal cortex (DLPFC) [6]. Recent evidence suggested that tDCS and serotonin enhance each other’s function. tDCS increases the release of serotonin mediated by the serotonin transporter [7]. In contrast, continuous enhancement of serotonin by antidepressants strengthens the long-term plasticity–like glutamatergic plasticity induced by tDCS [8]. In addition, tDCS was shown to modulate the activity of DLPFC [9, 10], which could modulate the hypoactive condition of the DLPFC in patients with depression. Furthermore, many patients with LLD display significant impairment in executive function, which is associated with a poor treatment response and prognosis in patients with LLD [11]. tDCS has been shown to improve executive function by stimulating the DLPFC region, which may cause an indirect improvement in depressive symptoms and the treatment response in patients with LLD [10]. Such findings support the theoretical treatment potential of tDCS as mono-therapy or augmentation therapy in patients with LLD.

#### Previous experience of tDCS as an intervention for depression

A growing number of studies have examined tDCS as a treatment intervention for depression. In a recent meta-analysis of six randomized controlled trials (RCTs) with 289 adult patients, tDCS was shown to be significantly superior to sham-control treatment in response, remission and improvement in depression. The left DLPFC was stimulated in most studies. The effect size was comparable with those reported for antidepressant drug treatment and for repetitive transcranial magnetic stimulation [12]. Controversial outcomes were noted in recent studies. The recent double-blind, placebo-controlled study compares the effect of tDCS and antidepressant, escitalopram. The result showed escitalopram was superior to tDCS despite no difference in the rates of response/remission [13]. Another large international RCT in adult patients with depression showed no difference in reducing depressive symptoms between active and sham stimulation in patients with unipolar or bipolar depression [14]. The conflicting results warrant further high-quality RCTs to determine the efficacy of tDCS. In addition, most studies have targeted the adult population. Few trials have been performed in older adults, and no RCT has been done in patients with LLD aged 60 or above. Only a small study investigated the effects of tDCS on executive function in older adults with Parkinson’s disease [15]; improvement in executive function was shown after tDCS was applied over the DLPFC region. It is essential to explore the effectiveness and tolerability of tDCS in older adults with depression.

#### Advantages of a new format of tDCS: HD-tDCS

A main drawback of traditional tDCS is the spatial crudity of its effect. The highest cortical current density may not occur directly under the target electrode [15], which probably alters the effects of tDCS and contributes to the conflicting results of existing tDCS studies. High-definition tDCS (HD-tDCS) was developed to address this problem. HD-tDCS is typically performed with more than two smaller electrodes. The most frequently used montage is the ‘4 × 1 ring set-up’, which uses a centre anode electrode surrounded by four return cathode electrodes. The density of the cortical field and spatial focality can be adjusted by changing the diameter of the ring of electrodes [16]. In addition to enhancing the spatial focality of stimulation, the increased effects of neuroplasticity may also be shown by longer-lasting after-effects, as in a previous study of HD-tDCS [17]. At the same time, the tolerability was found to be similar to that of traditional tDCS [18]. No HD-tDCS study of LLD or depression has yet been performed. Therefore, our team conducted a pilot study to test the effects and tolerability of HD-tDCS on subjects with LLD.

#### Results from our pilot study

An open-label pilot study was conducted by our team in 2018 [19]. 15 older adults aged 60 or above who have depression with residual depressive symptoms received five consecutive daily sessions of 20-min active HD-tDCS interventions weekly for 2 weeks. The results revealed that the HD-tDCS was effective in reducing the depressive severity and the remission rates, with a sustained effect at both the 1-month and 3-month follow-up. Pre-post improvements were seen in the overall cognitive functioning and in verbal fluency, but not in executive functioning. No adverse effects were observed. Nevertheless, this was an open-label study with a small sample size. A double-blinded RCT with an adequate sample size is warranted to confirm the efficacy of HD-tDCS as an augmentation therapy with antidepressants in patients with LLD.

### Objectives {7}

The main objective of this study is to determine the efficacy of a 2-week daily programme (10 sessions) of HD-tDCS to augment antidepressant therapy in subjects with LLD who had residual depressive symptoms despite adequate dosage and duration of antidepressant therapy.

Secondary objectives are to evaluate the HD-tDCS impact on the patients’ cognition (including the global cognitive functions, working memory, executive function and attention), anxiety symptoms, and daily functioning, after the 2-week daily programme (10 sessions) of HD-tDCS. The adverse effects of HD-tDCS and the patient’s tolerability will also be evaluated.

### Trial design {8}

The study described in this protocol is a RCT to evaluate the superiority of an active HD-tDCS programme for patients with LLD compared with the sham HD-tDCS programme. Forty-eight patients with LDD will be randomized (1:1) in the active HD-tDCS group or the sham HD-tDCS group.

## Methods: Participants, interventions and outcomes

### Study setting {9}

This 12-week double-blinded randomized sham-controlled trial will assess the efficacy of HD-tDCS as an augmentation therapy with antidepressants in subjects with LLD subjects who had residual depressive symptoms despite adequate dosage and duration of antidepressant therapy.

### Eligibility criteria {10}

The inclusion criteria are as follows:

1. Sixty years of age or above
2. Right-handedness, as determined by the Edinburgh Handedness Inventory (to homogenize neuroanatomical targeting)
3. Chinese ethnicity
4. Fulfil the criteria of major depressive disorder (single or recurrent episode) and in partial remission, defined by the 5th Edition of the Diagnostic and Statistical Manual of Mental Disorders (DSM-5)
5. Presence of mild to severe levels of depressive symptoms measured and defined by HAMD-17 scores ≥ 8 and ≤ 52 [20]
6. Suboptimal treatment response with at least one adequate antidepressant trial defined as full or best tolerated doses at least 6 weeks
7. Stable dosage of antidepressants or other treatments for depression in recent 4 weeks
8. Valid informed written consent

The exclusion criteria are as follows:

1. A DSM-5 diagnosis other than depressive disorders (e.g. bipolar and related disorders, schizophrenia spectrum and other psychotic disorders).
2. A Hong Kong Chinese version of the Montreal Cognitive Assessment (HK-MoCA) score below the second percentile according to the subject’s age and education level (to exclude subjects with existing dementia)
3. Substance use disorder
4. Active suicidal ideation or a suicide attempt within the past month
5. Concomitant unstable medical conditions or major neurological conditions
6. Significant communication impairment

### Who will take informed consent? {26a}

Informed consent is obtained from the patients before starting any trial-specific procedure. All participants are advised that participation in research is entirely voluntary and that they can withdraw their participation at any time. The psychiatrist does the first presentation of the study explaining with an information pamphlet detailing the purpose of the study, target audience, length and procedure of the treatment, possible risk factors, benefits of joining the study, summary of the function and effectiveness found in research related to using HD-tDCS as an augmentation treatment. Patients are given time to ask psychiatrists questions and express potential concerns. Patients will be given contact details of the clinical investigator for further enquires. Patients will also be given at least a month to decide whether to join the treatment post presentation of the study by psychiatrists given old adults with a suboptimal response may require some time to consult with families or to make a decision. The protocol is then explained again in detail by the clinical investigator before the signature of the informed consent by the patients.

### Additional consent provisions for collection and use of participant data and biological specimens {26b}

N/A, no biological specimens were collected in this study.

## Interventions

### Explanation for the choice of comparators {6b}

All eligible participants will be randomized to receive an intervention trial of HD-tDCS, either active intervention (a-HD-tDCS) or sham (s-HD-tDCS) as a control, in addition to their pre-existing antidepressant regimen, for 2 weeks with five sessions per week. A total of ten sessions HD-tDCS will be delivered, with each lasting 30 min.

### Intervention description {11a}

HD-tDCS intervention will be performed by formally trained and experienced investigators with hands-on experience in tDCS, who will not be involved in the data collection and analysis. There will be a demonstration and training session involving all staffs in delivering the treatment before the study. Videotape of the first session of each participant’s intervention would be done and reviewed by PI to ensure the quality. The device can also log down the parameter and energy used during the treatment. The intervention will be performed at either psychiatric outpatient clinics or psychiatric day hospitals, where the principal investigator or co-investigators serve on-site. The clinics and day hospitals are equipped with resuscitation facilities. Full-time nursing and support staff are available to handle medical emergencies.

#### High–definition transcranial direct current stimulation (HD-tDCS) group

The HD-tDCS will be administered by Starstim (Neuroelectrics). All participants will receive the treatment by using the same model of the device. The HD-tDCS device can be portable and controlled wirelessly via computer software developed by the manufacturer. The montages will be a ‘4 × 1 ring set-up’, which is the most commonly used HD-tDCS setting. The centre anode electrode is surrounded by four return cathode electrodes. The anode will be placed over the left DLPFC, which is located at F3 based on the 10/20 electroencephalogram system. The four cathodal electrodes will be placed at FC1, AF3, F7 and FC5 (Additional file 1). Conductive electrode gel will be applied on the scalp at all designated electrode stimulation areas. A cap appropriate for each participants’ head size will be used to ensure that the electrodes are secured in place. Impedance checks will be performed using the Starstim software before each treatment session. A 2-mA stimulation will be delivered for 20 min, with the current gradually increased and decreased over 30 s. The patients will be instructed to relax and remain motionless during the intervention. The administrator will closely monitor the impedance throughout each session and record any side effects experienced by the participants. The participants will be allowed 5 min of rest after the intervention and will be actively asked about any discomfort. Each session will last around 30 min, with a total of 10 sessions (two consecutive weeks of treatment for 5 days per week).

#### Sham–control HD-tDCS droup

The procedure for sham stimulation will be identical, except that the electrical current will be gradually ramped down to zero after the first 30 s, thus giving the same initial sensation of HD-tDCS. The stimulator will be programmed to switch the current on and off, so no intervention by the operator will be required. The computer will be placed behind the subjects’ heads so they cannot see the readout.

### Criteria for discontinuing or modifying allocated interventions {11b}

The intervention will be terminated upon the patients’ request.

### Strategies to improve adherence to interventions {11c}

To reduce the drop-out rate, each of the participants’ case medical officers will be alerted to remind the participants to attend all interventions and assessments. In addition, the benefits and side effects of HD-tDCS will be clearly explained during enrolment, and materials related to this study will be provided to both the participants and their caregivers. The participants and their caregivers will receive two reminder telephone calls (1 week and 1 day) before interventions and assessments. If a participant fails to attend an appointment, they will be called to rearrange the appointment. A direct hotline within office hours will be provided to the participants and their caregivers in case of any concerns or problems.

### Relevant concomitant care permitted or prohibited during the trial {11d}

All participants will be instructed to continue their pre-existing regimen of psychiatric medications, including antidepressants, benzodiazepines, hypnotics, for at least 4 weeks before the intervention until all post-intervention assessments are complete. Medication adherence will be recorded during each assessment.

### Provisions for post-trial care {30}

All participants will be continued to follow up by the case medical doctors in psychiatric outpatient clinics for their progress.

### Outcomes {12}

#### Baseline assessments

##### Diagnostic interview

Diagnoses will be confirmed with a semi-structured psychiatric diagnostic assessment using the validated Chinese-bilingual version of the Structured Clinical Interview for DSM Mental Disorders by an experienced psychiatrist according to DSM-V.

##### Demographic data

The subjects’ basic demographic data, including age, gender, years of education, place of birth, marital status, number of children, financial condition, family history of affective disorder and household income, will be collected upon study entry. Medical comorbidities will be assessed with the Cumulative Illness Rating Scale. The handedness of the participants will be evaluated using the Edinburgh Handedness Inventory – Short Form. Details of the subjects’ psychiatric history, including the age of onset of the first depressive episode, the number of relapse episodes and current medication and dosage, will also be recorded at the baseline assessment. The medical health history and treatment will be assessed by direct enquiry with the patients and confirmed with their electronic health record in the hospital authority.

##### Primary outcome

The primary outcome will be the change of depressive symptoms, the clinical response rate and the remission rate as measured with the HAMD-17 at 12 weeks after the completed intervention. HAMD-17 is a widely used and reliable measure of depressive symptoms [21]. Scores range from 0 to 52, with higher scores indicating more severe depression. A clinical response will be defined as a reduction of 50% or more in the HAM-D-17 score in old age adult patients. A HAMD-17 score of 7 or less will be used as an indicator of remission [20].

##### Secondary outcomes

1. Cognition

Global cognition will be measured using the Hong Kong Chinese version of the Montreal Cognitive Assessment (HK-MoCA) [22]. HK-MoCA is a cognitive screening test designed to assist health professionals to evaluate the cognitive functions of the patients. This encompasses the evaluation of visuospatial and executive functions, naming of object, memory, attention, language, abstraction, delayed recall and orientation. After completion of the test, the score in each domain will be documented and a total score out of 30 will be calculated for each participant. Working memory, executive function and attention will be measured by forward and backward digit span, the Stroop test, the category verbal fluency test and the Trail Making Test Parts A and B.

2. Anxiety symptoms

Anxiety symptoms which commonly co-existed in depression will be measured by the Hamilton Anxiety Rating Scale (HAMA). It is a widely used clinician-rated scale. The HAMA is composed of fourteen items, including anxious mood, tension, fears, insomnia, intellectual, depressed mood, somatic (muscular), somatic (sensory), cardiovascular symptoms, respiratory symptoms, gastrointestinal symptoms, genitourinary symptoms, autonomic symptoms, and behaviour at interview. Each item is scored on a scale of 0 (not present) to 4 (severe), with a total score range of 0–56, where < 17 indicates mild severity, 18–24 mild to moderate severity and 25–30 moderate to severe [23]. Higher marks represent more severe in anxiety symptoms.

3. Daily functioning

Instrumental activities of daily living will be assessed with the Hong Kong Chinese version of the Lawton Instrumental Activities of Daily Living Scale (IADL-CV) [24]. IADL-CV has been shown to be a valid and reliable tool for assessing an older person’s ability to live independently in the community. It encompasses the assessment of nine items, including telephoning, transportation, shopping, meal preparation, housework, handywork, laundry, medication and finance. For each domain, the patient’s IADL would be assessed by a four-point rating scale, with a total score ranging from 0 to 27. A higher score indicates a higher functioning level.

4. Adverse effects and risk indicators

A checklist of potential adverse effects associated with t-DCS administration will be generated from the available literature [25, 26]. The checklist will be used to monitor tolerability and adverse events in each session throughout the intervention (attached). Risk indicators such as suicidal risk or severe self-neglect that would necessitate immediate changes to treatment will be directly assessed according to the risks and needs.

### Participant timeline {13}

**Fig. 1:**
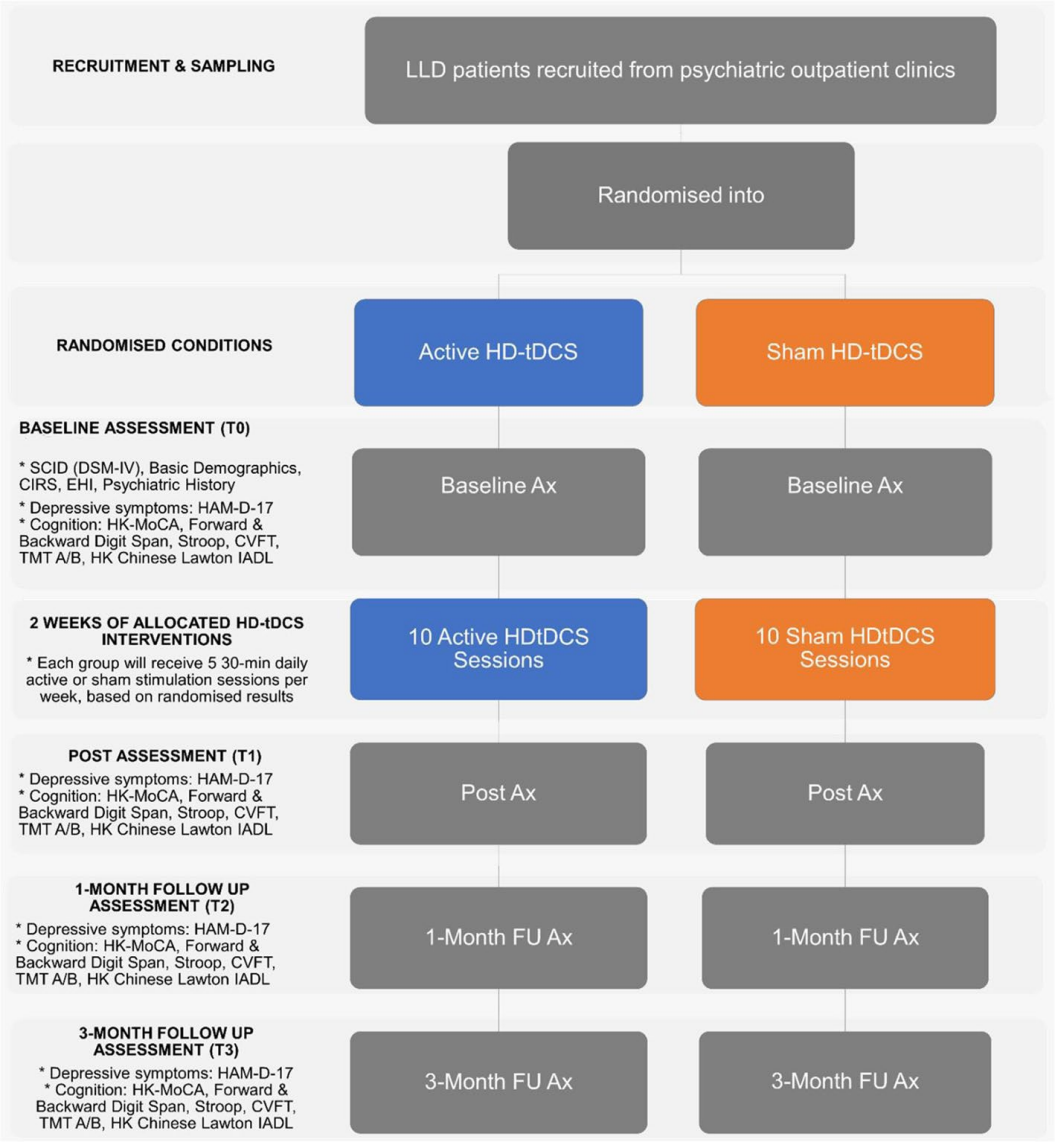
Flowchart of the current study

The participant timeline of the HD-tDCS intervention is shown in Fig. 1.

### Sample size {14}

The sample size was estimated using G*Power 3.1, based on the results of a tDCS meta-analysis of adult subjects by Kalu et al. [27]. In this study, active tDCS was found to be more effective than sham tDCS for the reduction of depression severity (Hedges’ g = 0.743). For 80% power and an alpha level of 5%, 48 patients (24 in each group) will be needed to detect group differences. To allow for a 20% drop-out rate, 58 patients (29 in each arm) will be recruited.

### Recruitment {15}

The recruitment procedure will begin after approval has been obtained from the Institutional Review Board. Psychiatrists from specialist outpatient clinics in the Hospital Authority of Hong Kong, the largest public medical institution, will be asked to refer older adults with depression but without active suicidal ideation or recent suicide attempt (within the past month) who do not show an adequate response to current antidepressant treatment (i.e. residual depressive symptoms). The clinician investigators will assess the potential subjects according to the exclusion and inclusion criteria as stated below. The diagnosis will be confirmed using the Structured Clinical Interview for Diagnostic and Statistical Manual Mental Disorders. Medical health history and treatment will be assessed by direct patient inquiry and confirmed using the patients’ electronic health records provided by the Hospital Authority. The subjects will be informed that the HD-tDCS treatment study will involve an off-label treatment, and the possible risks and side effects will be clearly explained.

## Assignment of interventions: allocation

### Sequence generation {16a}

Block randomization will be used in this study. Each block will consist of eight participants at a 1:1 ratio between the two groups. In the randomization procedure, each participant will be assigned a special number generated by a computer. These numbers will not be decoded until the intervention group is assigned.

### Concealment mechanism {16b}

An independent team member, who will not be involved in the enrolment, intervention, or assessments, will perform the randomization process. The participants and investigators responsible for assessment and data analysis will be blinded to the group allocation.

### Implementation {16c}

If participants are interested in participating in the treatment, psychiatrists will explain the study information and recruit them after screening out for exclusion criteria by collecting their contact information. Then, participants are contacted by telephone by the investigator. The investigator will inform the participants about the study and verify that the participants are aware of the implications of participation in a trial and propose an appointment for the initial evaluation. The participants will be given at least a month to consider whether or not to join the study. After the approval to join the study, participants are randomized to either the active HD-tDCS group or the sham HD-tDCS group by the research coordinator for subsequent intervention. Participants will then be contacted with the schedule and details of the treatment.

## Assignment of interventions: Blinding

### Who will be blinded {17a}

Trial participants and the trained investigator performing the interventions are double-blinded.

### Procedure for unblinding if needed {17b}

N/A. If there was a situation such as a medical need or emergency which required unblinding, the principal investigator will be informed and make the final decision.

## Data collection and management

### Plans for assessment and collection of outcomes {18a}

A trained clinician investigator who is not involved in the intervention or randomization procedure will perform the assessments. The primary and secondary outcomes will be assessed at baseline, immediately after the intervention, and 4 and 12 weeks after the intervention.

### Plans to promote participant retention and complete follow-up {18b}

To reduce the drop-out rate, each of the participants’ case medical officers will be alerted to remind the participants to attend all interventions and assessments. In addition, the benefits and side effects of HD-tDCS will be clearly explained during enrolment, and materials related to this study will be provided to both the participants and their caregivers. The participants and their caregivers will receive two reminder telephone calls (1 week and 1 day) before interventions and assessments. If a participant fails to attend an appointment, they will be called to rearrange the appointment. A direct hotline within office hours will be provided to the participants and their caregivers in case of any concerns or problems.

### Data management {19}

For each participant, clinical information regarding the diagnosis, description of clinical condition, severity of depression and the modalities of treatment and management are stored in the Clinical Management System (CMS) managed by the Hospital Authority (HA). Subsequently, formally trained research assistants will be responsible for collecting patient information and report in a data record form, first in a paper format and then electronically. The paper format data will be locked in a cabin in the office and electronic data is encrypted with passwords only available by the research assistants and data manager. The data manager will perform additional computerized consistency tests to detect the presence of non-standard, missing, aberrant or incoherent data. These tests will be executed regularly during the participants’ recruitment and monitoring. Each identified incoherence will be the subject of a request for clarification to the researcher. The data will be destroyed after 5 years commencing from the completion of the study.

### Confidentiality {27}

Personal information of participants will be collected at the initial interview, an identifier will be assigned to each enrolled participant. Information of participants will be encoded in an encrypted file with a password only available by the research assistants and data manager.

### Plans for collection, laboratory evaluation and storage of biological specimens for genetic or molecular analysis in this trial/future use {33}

N/A. No biological specimens will be collected in this study.

## Statistical methods

### Statistical methods for primary and secondary outcomes {20a}

The primary outcome measurement is HAMD-17. The effect power of intervention is measured by the change of HAMD-17. If the results showed a significant improvement in the HAMD-17 measurement, it represents the intervention is effective. Both clinical response rate and remission rate are also depending on the measurement of HADM-17. They are used as an indication for the real clinical impact of the intervention. A clinical response will be defined as a reduction of 50% or more in the HAM-D-17 score. A HAMD-17 score of 7 or less will be used as an indicator of remission. Therefore, a significant result in the area of either clinical response or remission rate will indicate the clinical impact of the intervention.

To examine the presence of any group differences in demographics and clinical profile between the treatment and the sham groups at baseline, chi-squared tests and *t*-tests will be performed for categorical and continuous variables respectively.

Two-way repeated measures analysis of variance (ANOVA) will be used to examine the effect of time and treatment conditions on the HAM-D-17 scores and other cognitive and IADL measurements across various assessment time points. The analysis would be adjusted to age, sex, medication type/dose, relapse episodes and other potential confounders. If both depressive symptoms and cognitive function improved, mediation analysis will be done to determine the cognitive function improvement is mediated by the improvement of depressive symptoms or vice versa.

### Interim analyses {21b}

Statistical analysis will be done only after the trial. The trial will be stopped if individual participants report serious adverse effects during the intervention, significant deterioration in mental state or physical condition. The decision will be made by the principal investigator.

### Methods for additional analyses (e.g. subgroup analyses) {20b}

Post hoc analyses will also be performed with Bonferroni corrected t-tests to investigate the improvements in all outcome variables in pairwise comparisons across various assessment time points. Statistical significance will be set at a *p* value < 0.05. Intention-to-treat last-observation carried-forward scores will be used for the analyses. All computations will be performed using SPSS for Windows, version 22.0.

### Methods in analysis to handle protocol non-adherence and any statistical methods to handle missing data {20c}

Participants will be allowed 2 non-consecutive missed visits and extra sessions will be performed to complete the total 10 sessions. Participants with more than 2 non-consecutive missed visits will be defined as non-compliance subjects. Further analysis would be done to compare the difference between compliance and non-compliance subjects.

### Plans to give access to the full protocol, participant-level data and statistical code {31c}

Protocol could be assessed by the public through the clinical trial register website. The study results will be published in an international journal. There are no plans to release the participant-level data.

## Oversight and monitoring

### Composition of the coordinating centre and trial steering committee {5d}

The principal investigator and other co-investigators, who are psychiatrists and neuroscientists, will be responsible to oversight and monitor the whole trial. Specialist psychiatrists in hospitals under Hospital Authority are responsible for the diagnosis of patients’ psychiatric disorders, recruiting patients and signing consents. If there are any disputes or logistic problems, the final decision will be made by the principal investigator.

### Composition of the data monitoring committee, its role and reporting structure {21a}

Data monitoring will be carried out by an independent data monitoring committee, which is composed of the research psychiatrist, a methodologist, a statistician and a data manager. The committee will ensure that all the data collection, storage and management procedures are carried out in accordance with the research protocol and the regulations. Committee members will keep track of and verify the data collection process. In case of any technical or procedural problems or uncertainties, the committee members are responsible for reporting to the principal investigator.

### Adverse event reporting and harms {22}

A checklist of potential adverse effects associated with t-DCS administration will be generated with reference to the available literature. During the stage of obtaining informed consent from the participants, all potential adverse effects associated with the use of t-DCS will be clearly listed and explained to the participants. In case there is any adverse effect, it will be immediately recorded in the clinical management system (CMS) and the data record form and the patient involved will be monitored by the principal investigator until resolution or stabilization. The patient condition will be closely monitored and the degree of seriousness of the adverse effect will be evaluated by the principal investigator who will then contact the trial promotor for further coordination.]

### Frequency and plans for auditing trial conduct {23}

N/A. The monitoring of the progress and quality of the trial will be conducted every month.

### Plans for communicating important protocol amendments to relevant parties (e.g. trial participants, ethical committees) {25}

Any amendments in the protocol will be discussed among all investigators and reported to the ethic committees responsible for the trial approval.

### Dissemination plans {31a}

Participants will receive a summary of their assessment reports after they finished the trial. The study result will be published in an international journal and presented in a relevant conference.

## Discussion

This is the first RCT that evaluates the effectiveness and tolerability of HD-tDCS in older adults with depression. Most studies tend to assess effectiveness in the adult population and only a few studies have evaluated the effects of tDCS on cognitive function in depression.

This randomized controlled trial aims to evaluate the effectiveness of HD-tDCS as augmentation therapy with antidepressants in ameliorating depressive symptoms in patients with LLD who had a suboptimal response to treatment. To this aim, the trial compares the effect of HD-tDCS and sham-control HD-tDCS on improving depressive symptoms in addition to the pre-existing antidepressant regimen. The depressive symptoms are operationalized into three levels, namely the change of depressive symptoms, the clinical response and the remission rate measured by HAMD-17. Secondary objectives are to examine the intervention impact on the cognitive function, anxiety symptoms, daily functioning and adverse effects at the end of the intervention. We also plan to detect any sustained effect by checking the changes at the 4th week and 12th week after the finished intervention.

The results from this trial will be useful for the management of patients with LLD because a new safe treatment option is needed to alleviate depressive symptoms for those showing suboptimal responses to conventional antidepressants. It is also expected that HD-tDCS is effective in improving cognitive function which is often significantly impaired in LLD. Based on its effect on neural plasticity and enhancement of serotonin and cognitive function, our study aims at confirming the efficacy of HD-tDCS as augmentation therapy for antidepressants in patients with LLD.

One of the limitations of the trial is the measurement of cognitive function by various tests e.g. HK-MoCA in which practice effects might come into play when evaluating the improvements in verbal fluency and overall cognitive ability. Although the same dosage and timing of antidepressants will be kept the same for at least 4 weeks prior to the study, the possibility of the effect from a change of medications cannot be excluded, which may lead to an overestimation of the effect by tDCS. Furthermore, the diversities of the clinical profiles might be potential confounding factors, including the types of antidepressants being used, the degree of suboptimal treatment response and treatment resistance, or the duration of the depressive symptoms. Subgroup analysis will be performed to eliminate the bias from confounding factors and enhance the validity of the trial.

## Trial status

Protocol version: 23 February 2022, Version n˚1.

Date of recruitment: 1st Sep 2020.

End of recruitment: estimated to be Jan 2023.

## Supplementary Information

**Additional file 1.** HD-tDCS setting of electrode annotations.

**Additional file 2.** HDtdcs Adverse Effects Checklist.

## Data Availability

Data will be available upon request to the investigator.

## Abbreviations

a-HD-tDCS: Active high-definition transcranial direct current stimulation
DLPFC: Dorsolateral prefrontal cortex
DSM-5: Diagnostic and Statistical Manual of Mental Disorders (DSM-5)
HAMA: Hamilton Anxiety Rating Scale
HAMD: Hamilton Depression Rating Scale
HD-tDCS: High-definition transcranial direct current stimulation
HK-MoCA: Hong Kong Chinese version of the Montreal Cognitive Assessment
IADL-CV: Hong Kong Chinese version of the Lawton Instrumental Activities of Daily Living Scale
LLD: Late-life depression
RCTs: Randomized controlled trials
s-HD-tDCS: Sham high-definition transcranial direct current stimulation
tDCS: Transcranial direct current stimulation
