# Supplementary figures and images for: High-definition transcranial direct current stimulation (HD-tDCS) as augmentation therapy in late-life depression (LLD) with suboptimal response to treatment—a study protocol for a double-blinded randomized sham-controlled trial
Source: Trials. 2022 Oct 28;23:914. doi: 10.1186/s13063-022-06855-z (PMC9617316; doi:10.1186/s13063-022-06855-z)

Mount  
8 Mount

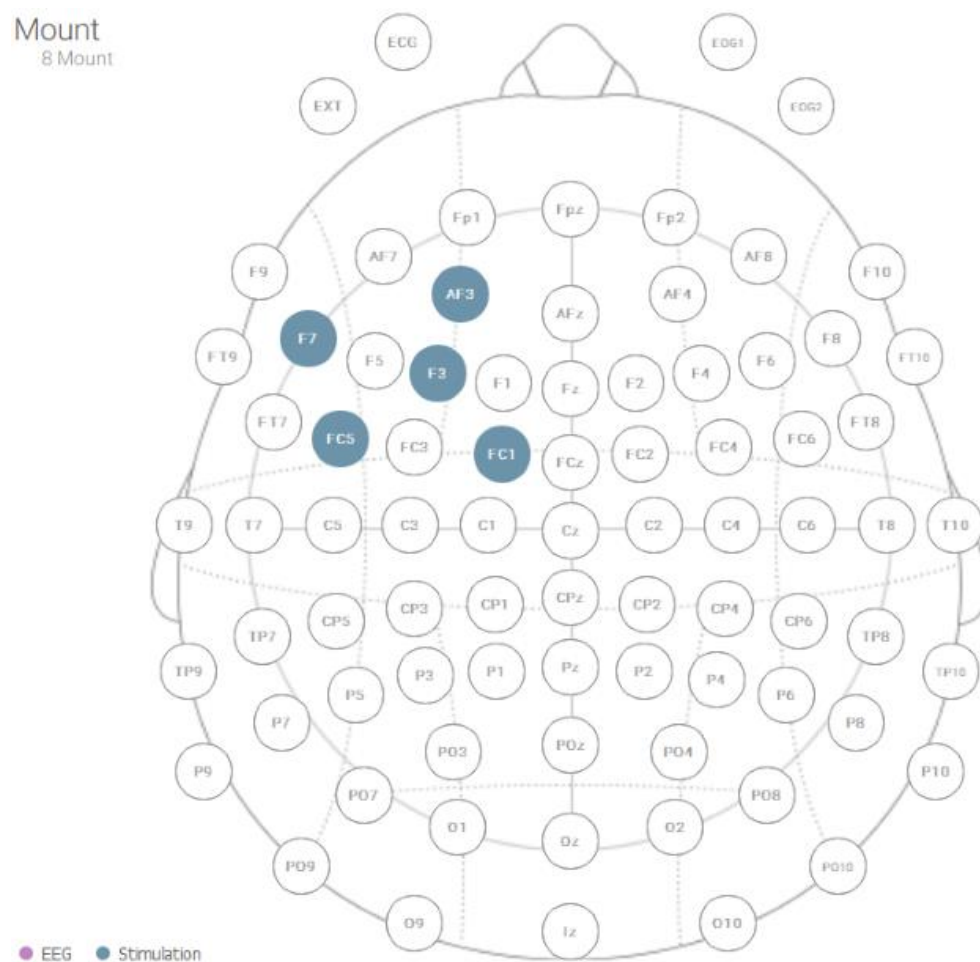

Supplement: Supplementary file 1 — Additional file 1. HD-tDCS setting of electrode annotations. [file 13063_2022_6855_MOESM1_ESM.pdf]
