# Supplementary material for: High-definition transcranial direct current stimulation (HD-tDCS) as augmentation therapy in late-life depression (LLD) with suboptimal response to treatment—a study protocol for a double-blinded randomized sham-controlled trial
Source: Trials. 2022 Oct 28;23:914. doi: 10.1186/s13063-022-06855-z (PMC9617316; doi:10.1186/s13063-022-06855-z)
Supplement: Supplementary file 2 — Additional file 2. HDtdcs Adverse Effects Checklist. [file 13063_2022_6855_MOESM2_ESM.pdf]

HDtdcs Adverse Effects Checklist

**Title of project**	高清晰度腦部直流電刺激對老年抑鬱症療效之研究										
**Participant No**											
	**Week 1**										
**After session**	**1**		**2**		**3**		**4**		**5**		**After session**
**Date**	**/ /2020**		**/ /2020**		**/ /2020**		**/ /2020**		**/ /2020**		**Date**
	**Severity**	**Related to HDtdcs?**	**Severity**	**Related to HDtdcs?**	**Severity**	**Related to HDtdcs?**	**Severity**	**Related to HDtdcs?**	**Severity**	**Related to HDtdcs?**	
Tingling*											Tingling*
Itching*											Itching*
Skin redness*											Skin redness*
Burning sensation*											Burning sensation*
Headache*											Headache*
Neck pain*											Neck pain*
Scalp pain*											Scalp pain*
Numbness											Numbness
Fatigue after stimulation/ Sleepiness*											Fatigue after stimulation/ Sleepiness*
Dizziness											Dizziness
Nausea											Nausea
Trouble concentrating*											Trouble concentrating*
Acute mood change*											Acute mood change*
A phosphene (i.e. a brief flash of light) at the start of stimulation											A phosphene (i.e. a brief flash of light) at the start of stimulation
Others (Please specify)											Others (Please specify)
Remarks											Remarks

Severity?	1	absent	2	mild	3	moderate	4	severe		
AE related to HDtdcs?	1	none	2	remote	3	possible	4	probable	5	definite

*also in Brunoni et al 2011 Tdcs AE qnr

HDtdcs Adverse Effects Checklist

**Title of project**	高清晰度腦部直流電刺激對老年抑鬱症療效之研究
**Participant No**	

	**Week 2**										
**After session**	**1**		**2**		**3**		**4**		**5**		**After session**
**Date**	**/ /2020**		**/ /2020**		**/ /2020**		**/ /2020**		**/ /2020**		**Date**
	**Severity**	**Related to HDtdcs?**	**Severity**	**Related to HDtdcs?**	**Severity**	**Related to HDtdcs?**	**Severity**	**Related to HDtdcs?**	**Severity**	**Related to HDtdcs?**	
Tingling*											Tingling*
Itching*											Itching*
Skin redness*											Skin redness*
Burning sensation*											Burning sensation*
Headache*											Headache*
Neck pain*											Neck pain*
Scalp pain*											Scalp pain*
Numbness											Numbness
Fatigue after stimulation/ Sleepiness*											Fatigue after stimulation/ Sleepiness*
Dizziness											Dizziness
Nausea											Nausea
Trouble concentrating*											Trouble concentrating*
Acute mood change*											Acute mood change*
A phosphene (i.e. a brief flash of light) at the start of stimulation											A phosphene (i.e. a brief flash of light) at the start of stimulation
Others (Please specify)											Others (Please specify)
Remarks											Remarks

Severity?	1	absent	2	mild	3	moderate	4	severe		
AE related to HDtdcs?	1	none	2	remote	3	possible	4	probable	5	definite

*also in Brunoni et al 2011 Tdcs AE qnr
